# Stem Cell Therapy for the Heart: Blind Alley or Magic Bullet?

**DOI:** 10.1007/s12265-016-9708-y

**Published:** 2016-08-19

**Authors:** Arne A. N. Bruyneel, Apurv Sehgal, Sophia Malandraki-Miller, Carolyn Carr

**Affiliations:** 1 Department of Physiology, Anatomy, and Genetics, University of Oxford, Oxford, UK; 2 University of Oxford Medical School, Oxford, UK

**Keywords:** Heart disease, Stem cell therapy, Cardiac stem cells, Regenerative medicine

## Abstract

When stressed by ageing or disease, the adult human heart is unable to regenerate, leading to scarring and hypertrophy and eventually heart failure. As a result, stem cell therapy has been proposed as an ultimate therapeutic strategy, as stem cells could limit adverse remodelling and give rise to new cardiomyocytes and vasculature. Unfortunately, the results from clinical trials to date have been largely disappointing. In this review, we discuss the current status of the field and describe various limitations and how future work may attempt to resolve these to make way to successful clinical translation.

## Introduction

Heart disease is one of the leading causes of death worldwide. The human heart, in contrast to various other organs like the liver, skin and gut, is unable to cope with severe tissue damage. Stem cells have been suggested as a tool to regenerate damaged contractile and vascular tissue and/or prevent adverse remodelling post-myocardial infarction (MI).

Most life years lost due to death and disability in the Western world arise from non-communicable diseases, such as cancer and cardiovascular disease (CVD). While the risk of death by CVD is decreasing in developed countries, it is becoming more prevalent in developing and transitional countries, with 80 % of CVD-related deaths occurring in low- or middle-income countries, partly as a result of increasing longevity, society and lifestyle changes [1–3]. Despite significant improvements in treatment strategies and survival rates, acute coronary syndromes account for half of all cardiovascular deaths, with 18 % of men and 23 % of women, older than forty, dying within a year of a first MI [2, 4]. Moreover, over time compensatory mechanisms may not be sufficient, leading to heart dilation and HF in the majority of patients. This condition has a dire prognosis of approximately 50 % mortality 5-years post-diagnosis [5].

Given the complex pathophysiology of heart failure and adverse remodelling of the heart tissue, therapeutic strategies should aim to both alleviate symptoms and attenuate further adverse ventricular remodelling. Although there has been considerable improvement in survival of some patient groups suffering from HF, there is no curative treatment available other than transplantation. However, donor organs are sparse and transplanted patients are required to take lifelong immunosuppressive drugs [6]. Alternatively, heart pumping can be supported by implantation of a mechanical ventricular assist device, either used as a bridge to transplantation or as destination therapy [7, 8].

Since the loss of cardiomyocytes underpins the pathophysiology of myocardial infarction (MI) and initiates the transition to HF, stem cell therapy (SCT) has been proposed as a potential therapeutic strategy, as these cells have the potential to form new contractile tissue.

## The Heart is not a Post-Mitotic Organ

Whether or not the heart is a terminally differentiated organ or has a stem cell population has been a contentious issue [9–11]. Many groups have tried to determine the cardiomyocyte turnover rate in the heart, with rates around 1 % being most commonly reported [12–14]. Bergmann et al. [13] determined cardiomyocyte turnover using the incorporation of carbon-14 ( ^14^C), from nuclear bomb tests, in genomic DNA, and demonstrated that cardiomyocyte (CM) DNA synthesis continues throughout life at annual rates ranging from 0.5 to 2 %, decreasing with age. A follow-up study by Senyo et al. [12], using ^15^N imaging mass spectrometry, reported a similar annual rate of about 0.76 % per year in the young adult mouse, and again the rate declined with age. Interestingly, they also observed an increase after myocardial injury in the border region, as was reported previously [11]. In addition, Mollova et al. [14] also observed cardiomyocyte cytokinesis in human infants, which decreased with age and was absent in adults.

There is considerable confusion as to where these new cardiomyocytes arise from, with at least three potential sources: (a) pre-existing cardiomyocytes, (b) resident stem or progenitor cells, or (c) circulating stem or progenitor cells. Bergmann’s study did not determine the origin of these newly derived cardiomyocytes [13]. In zebrafish, existing cardiomyocytes were shown to contribute to regeneration post-myocardial injury [15]. Similarly, Senyo et al. [12] demonstrated that new cardiomyocytes were generated from pre-existing cardiomyocytes and that cardiac progenitors played an insignificant role in myocardial homeostasis in health and disease. In contrast, other studies identified resident stem cell populations with the capability to give rise to the cardiomyocyte lineages of the heart [16, 17] or claimed that circulating cells contribute to myocytes and blood vessels [18].

## Stem Cell Therapy for Cardiovascular Disease

During development, stem cells form the organs and tissues in the body, and by the time the foetus is fully formed most of these more potent stem cells have disappeared. Adult organisms contain adult progenitors to enable tissue homeostasis. The rationale for using stem cells for heart disease treatment is that these cells might give rise to new cardiomyocytes and blood vessels, to replace the tissue lost post-MI.

Embryonic stem cells and multiple sources of adult stem cells have been suggested as suitable candidates for regeneration post-MI or HF and have been tested in animal models and in the clinic, as discussed in the following sections.

### Bone-Marrow Derived Cells

Historically, the first studies which tried to use stem cells to repair damaged heart tissue involved skeletal myoblasts or bone marrow (BM)-derived stem cells, because of availability and existing experience in bone-marrow transplantations.

Infusion or injection of BM cells was shown to regenerate myocardium in vivo and had beneficial effects on cardiac function [19, 20]. In addition, bone marrow-derived mesenchymal stem cells (BM-MSCs) were shown to be able to differentiate in vitro to cardiomyocytes [20, 21]. However, later studies questioned the cardiomyogenic potential of BM-derived cells [22], suggesting that the infused cells either act through paracrine signalling, fuse with resident CMs or differentiate to a mature haematopoietic lineage [22–24]. There has now been more than a decade of clinical experience with bone marrow cells for the treatment of acute myocardial infarction (AMI), HF or angina. The latest Cochrane review concluded that there is currently insufficient evidence for a beneficial effect of BM cell therapy for AMI patients [25], whereas a recent trial sequential analysis suggested that current randomised controlled trials which administering autologous BM-derived cells to HF patients offered a reduction of the risk of mortality and hospitalisation for HF [26]. The results of large phase-3 trials will likely shed more light on the ability of BM-derived cells to provide therapeutic benefits. Three phase-3 studies are currently ongoing and may be expected to report results within the next couple of years: BAMI (NCT01569178), CHART-1 (NCT01768702) and the DREAM-HF trial (NCT02032004).

### Skeletal Myoblasts

Skeletal muscle is easily accessible and contains myoblasts that are resistant to ischaemia and proliferate to repair or replace damaged or old muscle tissue [27, 28]. This led various groups to explore the feasibility of applying skeletal myoblasts for cardiac regeneration.

Myoblast transplantation improved cardiac function in animal studies [28] and milieu-dependent differentiation of myoblast satellite cells into cardiac-like muscle cells was observed in some studies [29]; however, this observation was heavily disputed by others [27]. Nonetheless, this technology was soon translated into clinical trials and, although some patients developed ventricular arrhythmias, phase-I studies demonstrated safety and promised hope for heart failure patients. However, they failed to fulfil the promised results in phase-II clinical studies [30, 31]. Since myoblasts do not differentiate to form cardiomyocytes in significant numbers, and the transplanted cells failed to gain electromechanical coupling with the host tissue, interest in these cells diminished [32].

### Embryonic and Induced Pluripotent Stem Cells

Embryonic stem (ES) cells are typically derived from the inner cell mass of pre- or peri-implantation mammalian embryos. These cells can give rise to all three germ layers and thus differentiate into all tissues. However, they have considerable risk of rejection since they are not autologous and entail ethical issues as they are derived from fertilised eggs. Induced pluripotent stem (iPS) cells are ES cell-like and can be derived from the somatic cells of patients, which makes them a potential autologous source [33]. Reprogramming to form iPS cells from somatic cells was originally accomplished by overexpression of pluripotency-related transcription factors: OCT4, SOX2, KLF4 and MYC using a retroviral approach. More recently, significant improvements have made the process more efficient and the use of integrating vectors obsolete [34].

There are very few clinical trials using ES-derived therapies for regeneration. For the heart, the feasibility of using embryonic stem cell-derived cardiomyocytes (ES-CMs) on a clinical scale was demonstrated in a non-human primate model [35], making progress towards clinical translation. Recently, Menasché et al. [36] started a clinical study with ES-derived cardiac progenitor cells embedded in a fibrin scaffold. It is still too early to assess the therapeutic benefit and safety, but the initial results are promising. However, care should be taken in clinical translation, as these stem cells have the potential to form tumours.

### Endogenous Cardiac Stem Cells

More recently, multiple ways to isolate or identify endogenous or resident cardiac progenitor cells (CPCs) have been reported (c-kit [16], Sca-1 [37], ALDH [38], Bmi1+ cells [39], side population [40], epicardial [41] and cardiospheres [42]), and the longstanding theory that the heart is a terminally differentiated organ was abandoned [13]. Typically, these cells express CM transcription factors, but lack contractile protein expression which they may acquire after differentiation to CMs [37].

C-kit cardiac stem cells (CSCs) are the most studied cell type but are also the most disputed. Beltrami et al. [16] identified a Lin- c-kit+ population which could give rise to cardiomyocytes, smooth muscle and endothelial cells and showed beneficial effects after injection in an experimental MI animal model. More recently, Ellison et al. [43] postulated that c-kit+ cells are both necessary and sufficient for cardiac regeneration in a model of diffuse myocardial damage, demonstrating both that the cells successfully contributed to the regeneration post-injury and that ablation of the resident cells abolished the functional recovery, which could be rescued by application of exogenous cells. The results were contested because of potential issues with the experimental methods used [44]. Du et al. [45] reported that c-kit+ cells contributed to the formation of new CMs in the neonatal, but not the adult, heart whilst van Berlo et al. [46] reported that endogenous c-kit+ cells give rise to cardiomyocytes within the adult heart, at a level of approximately 0.008 %, but contributed to the development of cardiac endothelial cells. This was also challenged, again citing issues with methodology [47], but the findings were independently confirmed by Sultana et al. [48].

Sca-1 is a cell surface protein involved in cell signalling and adhesion and Sca-1 ^+^ resident cardiac stem cells were first described by Oh et al. [37] in 2003, as small interstitial cells adjacent to the basal lamina in mouse hearts. Uchida et al. [17] demonstrated that Sca-1+ CSCs contribute to the generation of CMs during normal ageing and after injury and Sca-1 positive cells improved cardiac function after administration post MI [49]. Similarly, Sca-1 deletion resulted in impaired cardiac function with ageing, and hypertrophy [50] and interestingly, Sca-1 KO mice also had reduced resident c-kit CPCs and reduced CPC migration post-MI. Although Sca-1 is absent in humans, a ‘Sca-1 like’ population of cells can be isolated from the human heart by selection using the murine antibody [51].

Closely related to the Sca-1 cells are the cardiac side population (SP) cells which were identified based on their ability to extrude Hoechst 33342 using the Abcg2 transporter [40]. About 80−90 % of the SP are Sca-1 ^+^, whereas only about 1 *%* of Sca-1 ^+^ cells are SP cells [52]. A recent paper by Doyle et al. [53] suggested that the SP cells form cardiomyocytes, endothelial cells and vascular smooth muscle cells during cardiac embryogenesis and contribute to the development of new vasculature, but not cardiomyocytes, post-MI.

Finally, Messina et al. [42] isolated and expanded another population of cardiac stem cells, named cardiosphere-derived stem cells (CDCs). These cells can be isolated from patient biopsies and the effect of comorbidities on these cells has been assessed [54–57]. CDCs were shown to differentiate into cardiomyocytes and endothelial cells in vitro, in response to 5’-azacytidine or transforming growth factor stimulation [57, 58]. Additionally, CDCs have been shown to have beneficial effects after transplantation in experimental infarction models [54, 59]. Most recently, Gallet et al. [60] demonstrated that CDCs were able to ameliorate heart failure with preserved ejection fraction in an experimental rat model by decreasing fibrosis and inflammation.

Some effort has been made to assess how these populations differ and how they relate to the cells in the cardiac stem cell niche. Dey et al. [61] applied microarray-based transcriptional profiling on three CSCs populations (ckit+, Sca-1+ and SP) in mice, which revealed that the ckit+ population differed from Sca-1+ and SP cells, with Sca-1+ being the most similar to CMs. In addition, based on transcriptome data published by others, they concluded that CDCs were most closely related to BM-MSCs. Noseda et al. [62] performed single-cell qRT-PCR profiling on Sca-1 cells and demonstrated that PDGFR *α* is superior to the SP phenotype for demarcating cardiac transcription factor expressing cells.

Clinical trials have used or are using a range of endogenous cardiac stem cells. In 2011, the Anversa group published the promising results of the phase-I Stem Cell Infusion in Patients with Ischemic cardiOmyopathy (SCIPIO) trial using c-kit ^+^ cells [63]. Patients with a history of post-MI cardiac dysfunction were treated with either 0.5 or 1 million c-kit CSCs. However, in 2014, *The Lancet* published an expression of concern with respect to the integrity of the clinical trial [64]. CDCs also underwent phase-I testing, in the CArdiosphere-Derived aUtologous stem CElls to reverse ventricUlar dySfunction (CADUCEUS) trial, on 17 patients with left ventricle (LV) dysfunction post-MI where 12.5 to 25 million cells were infused intracoronary (IC). The initial results demonstrated safety, and a reduction in scarring after myocardial infarction, although without significant improvement in ejection fraction (EF) [65]. HF patients were treated with CSCs enriched for ES and mesenchymal stem cell (MSC) markers in the Autologous human cardiac-derived stem cell to treat ischemic cardiomyopathy (ALCADIA) trial [66] and the injection sites were covered by a biodegradable gelatin hydrogel sheet containing 200 *μ*m basic fibroblast growth factor (bFGF). The ALCADIA trial demonstrated safety, but larger trials will be required to assess the therapeutic potential of this cell plus biomaterial approach.

CSCs have also been tested in the transcoronary infusion of cardiac progenitor cells in patients with single (TICAP) trial [67], where children with the congenital heart defect, hypoplastic left heart syndrome (HLHS) were treated with CDCs. Safety of the procedure was demonstrated, and the stem cell-treated patients had improved right ventricular ejection fraction (RVEF) at 18 months of follow-up, in contrast to control patients.

In summary, current clinical trials using CSCs have demonstrated safety and hope for therapeutic efficacy. Larger randomized controlled trials will be required to assess efficacy, ideal cell dose, time, frequency and route of administration.

## Mechanism of Action

**Fig. 1:**
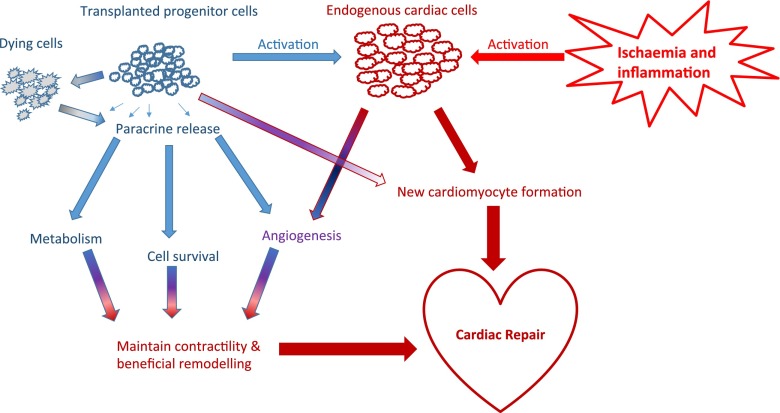
Mechanism of action of stem cell therapy post-MI. The transplanted cells and their progeny are activated by the local inflamed and ischaemic milieu. The transplanted cells can exercise beneficial effects on the heart directly by differentiation or indirectly by the secretion of paracrine factors. Similarly, the transplanted cells may recruit and activate endogeneous cells from the heart or from elsewhere within the body, which may differentiate or induce further paracrine signaling. In addition, the death of the transplanted cells may modulate the inflammatory environment

It remains unclear which mechanism(s) lead to the beneficial effects seen in both animal models and clinical trials. Three non-mutually exclusive mechanisms have been proposed: (a) the transplanted cells and/or their progeny aid in the regeneration, (b) factors produced by the infused cells stimulate endogenous regeneration or alter the tissue’s response to injury and (c) the death of the infused cells alters the body’s response to injury (Fig. 1).

### Direct Regeneration

Given the significant loss of contractile tissue and the generation of scar tissue, de novo remuscularisation is considered the magic bullet for HF treatment. Multiple studies have presented evidence in favour of exogenous or endogenous stem cell contribution to the generation of new cardiomyocytes [43, 59]. The study by Chong et al. [35] demonstrated that a significant proportion of the infarcted monkey heart could be remuscularized using ES-CMs after injection of one billion cells, suggesting that this similarly would be possible in the human heart. However, in most cases, only a very few de novo cardiomyocytes can be identified and cell retention post injection or infusion is typically very low [68], suggesting that direct remuscularisation likely only has a small contribution to any beneficial effect.

### Paracrine Effects

Paracrine signalling involves the release of effector agents capable of inducing a regenerative or protective response. These agents include inter alia cytokines, growth factors, micro RNAs (miRNAs) and secreted extracellular vesicles like exosomes which can contain proteins and RNA molecules. The paracrine signalling molecules vary with the type of stem cell, and their effects include preventing death and adverse remodelling, maintaining cardiac contractility and metabolism and promoting neovascularisation and cardiac regeneration [69].

Chimenti et al. [70] quantified the relative contribution of paracrine signalling and direct regeneration in the immune deficiency mouse model after injection of human CDCs, by quantifying the proportion of cells that were of human origin versus the overall improvement in cardiac parameters. The study revealed that paracrine interactions significantly outweighed direct regeneration, and even though the capillary density doubled in CDC-treated mice, only 10 % of new vessels were of human origin. Similarly, treated mice had a higher proportion of viable myocardial tissue, but only 12 % of the myosin heavy chain-positive cells in those areas was of human origin. Interestingly, they reported the development of foci of murine c-kit+ cells near human CDCs, and the recruitment of nkx2.5+ cells in the infarcted area.

MSCs have been shown to secrete anti-apoptotic factors and to modulate the immuno-inflammatory response post-MI [71]. The phosphoinositide 3-kinase (PI3K)-Akt pathway plays a central role in pro-survival signalling [72]. Insulin-like growth factor-1 (IGF-1) is a potent activator of the Akt pathway leading to survival of CMs and is secreted by MSCs and endogenous CSCs [73]. In a model of heart failure with preserved ejection fraction in which CDC SCT reduced scaring and inflammation, it was proposed that CDCs released exosomes containing miRNA and modulated gene expression [60]. Du et al. [74] reported that transplantation of MSCs inhibited the activity of NF- *κ*B, attenuated the production of pro-inflammatory proteins including tumor necrosis factor- *α* and interleukin-6 (IL-6) and increased the expression of the anti-inflammatory protein interleukin-10 (IL-10) in peri-infarct myocardium. Furthermore, Ohnishi et al. [75] demonstrated that MSC-conditioned medium upregulated the expression of anti-proliferation genes and downregulated the expression of collagen I and III in cardiac fibroblasts.

Paracrine induction of neovascularisation involves mediators such as vascular endothelial growth factor (VEGF) and bFGF which are secreted by a variety of cells, including CDCs and MSCs [69, 76]. Exogenous stem cell transplantation may also activate resident CSCs and stimulate cardiomyocyte replication via paracrine signalling. Linke et al. [77] found that intramyocardial injection of hepatocyte growth factor (HGF) and IGF-l induced formation of new myocytes and blood vessels. Similarly, Yoon et al. [78] reported that a population of BM-derived stem cells could induce endogenous and exogenous cardiomyogenesis. The cytokine stromal cell-derived factor-1 (SDF-1) has also been shown to promote cell survival, endogenous stem cell recruitment, and vasculogenesis [79].

Taken together, transplanted cells have the potential to secrete a large variety of paracrine factors, and these affect multiple pathways with overlapping effects leading to protection post-MI simultanuously.

### The Dying Stem Cell Hypothesis

Thum et al. [80] hypothesized that the beneficial effect of stem cell transplantation could be explained by the modulation of local immune reactions in response to apoptosis of the infused cells. Dying cells release danger signals which may trigger immune responses, but the mode of cell death differs between the native heart cells and the injected stem cells. Necrotic cell death is the major contributor to cell death in the infarcted heart [81], whereas apoptotic cells inhibit inflammation [82].

Stressed peripheral blood mononuclear cells have been shown to enhance angiogenesis and wound healing, resulting in tissue repair through paracrine signalling pathways [83]. Burt et al. [84] demonstrated that irradiated and mitotically inactivated ES cells were capable of improving myocardial function after injection into the infarcted heart, to the same extent as non-irradiated ES cells. They saw minimal cell engraftment and no improvement in cardiac function after injection of conditioned medium, suggesting that the beneficial effect was most probably dependent on the transitory presence of the cells. Interestingly, injection of mouse embryonic fibroblasts cells did not ameliorate cardiac function post-MI, suggesting that the type of dying cell might be pivotal to the beneficial effect observed.

In conclusion, although the underlying mechanisms of cardiac cell therapy are still unclear, current data suggests that paracrine mechanisms, either as a result of factor secretion or stem cell death, contribute the most.

## Strategies for Improving the Therapeutic Efficacy of Cell Therapy

Cellular retention directly relates to the beneficial therapeutic outcomes observed [85]. Cells are typically delivered to the damaged area by either vascular infusion or direct myocardial injection, neither of which is particularly efficient. Indeed, Pons et al. [86] reported that 90 % of the injected stem cells were lost within the first day, and 99 % in the first week. There are many mechanisms leading to poor cell survival and retention [87] including cell death or limited self-renewal in the harsh microenvironment of hypoxia, inflammation, oxidative stress and as a result of the continuous compressive mechanical stress in the heart which pushes cells outwards from the injection sites. Hence, strategies aimed at improving retention of infused stem cells within the the heart are currently being investigated [87].

**Fig. 2:**
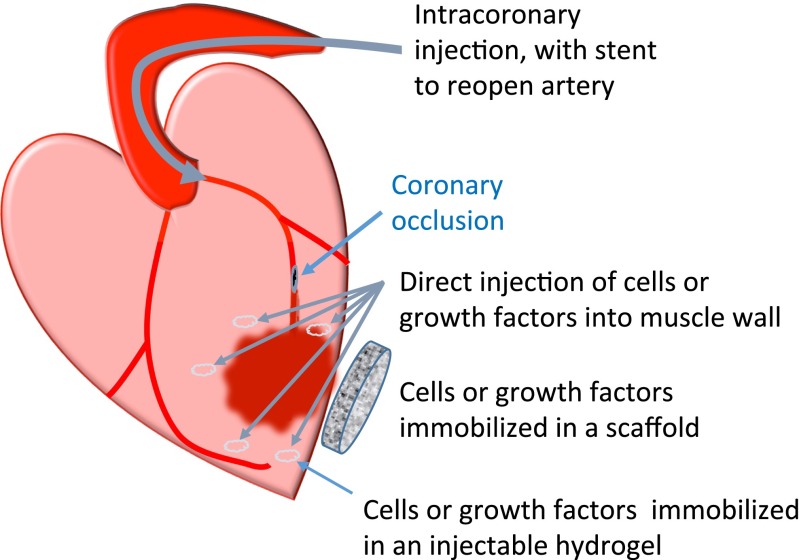
Cell or paracrine factor delivery methods. Cells or paracrine factors may be delivered via the coronary vasculature or injected directly into the heart muscle. They may be immobilized in an injectable hydrogel or in a scaffold attached onto the epicardium

### Devices for Delivery

Cells can be delivered into the heart via different routes: IC, intramyocardial (IM) and intravenous (IV) [88] (Fig. 2). In preclinical studies, IV delivery or epicardial injections into the infarct border zone are more common. In large animal studies or clinical trials, cells can also be injected into the endocardial surface of the myocardium or infused directly into the coronary arteries. IM injection has been shown to be superior to IV infusion, although more invasive and technically challenging [89].

Regardless, cell retention remains very low within the heart, and hence, additional research for alternative devices is necessary. Dib et al. [90] investigated the effect of balloon inflation on the viability of the infused cells, and suggested that multi-lumen catheters are superior. Behfar et al. [91] modelled alternative needle designs to the conventional straight or helical needles with an end-hole. Their optimum design of a curved needle with side holes gave a 3-fold increase in retention compared with a straight needle with end-hole. Soubihe et al. [92] also proposed a novel injection needle, with a blunt tip and multiple 0.5 mm diameter holes and with a brush-mandrill to make micro-lesions and prime the cardiac tissue to receive the cells.

### Improvement of Cell Homing to the Heart

Methods that make infused cells home more efficiently to the area of need could make direct injection obsolete. Cell adhesion markers, signal molecules such as chemokines, growth factors or hormones play prominent roles in the recruitment of cells to target tissues [93]. To improve homing, either the therapeutic cells could be modulated to be more responsive to the endogenous signals, or the target tissue could be encouraged to produce more signal. One of the most thoroughly studied chemo-attractants is SDF-l which has been shown to attract circulating progenitor cells to injured or ischaemic tissues via its receptor C-X-C chemokine receptor 4 (CXCR4) [94].

#### Cell Homing Strategies

The therapeutic cell population can be primed for more effective homing by conditioning, chemical treatment or genetic modification. Exposure of MSCs to 1 % oxygen upregulated CXCR4 expression in a hypoxia-inducible factor (HIF)-dependent manner and increased the in vitro migration to SDF-l [95]. Similarly, BM-MSC treatment with hypoxia-mimetics upregulated CXCR4 expression [96]. However, human MSCs overexpressing CXCR4 did not have improved cell migration, suggesting that additional mechanisms might be required for effective homing [97].

Glycoengineering of the cell surface of the infused stem cells with physiological selectin-ligands has also been suggested as a method to enhance engraftment. Lo et al. [98] validated two glycoengineering protocols in a porcine ischaemia/reperfusion (I/R) model and demonstrated homing of the modified stem cells to sites of I/R in the heart.

#### Tissue Recruitment Strategies

Tissue-specific recruitment strategies, for example using biomaterials carrying signaling molecules, are considered in situ tissue engineering strategies [99]. The delivered signalling molecules could either target the endogenous stem cells or stimulate stem cells in the BM. SDF-l can be delivered to increase cell homing to the tissue, but it is rapidly degraded. Segers et al. [100] delivered protease-resistant SDF-l into the infarct border area, using self-assembling peptide nanofibers, and noted improved cardiac function after MI. Similarly, delivery of MSCs overexpressing SDF-l to the ischemic myocardium facilitated repair by the recruitment of progenitor cells [101]. However, a recent phase-II trial using a single dose of SDF-l gene therapy failed to meet its primary endpoint, although it did show beneficial effects in one of the patient subgroups [102]. Erythropoietin (EPO) supplementation prevented LV-dilatation and deterioration of cardiac function post-MI, attributed to increased capillary growth as a result of VEGF expression by the myocardium and EPO-induced mobilisation of endothelial progenitor cells (EPCs) from the BM with homing to the cardiac microvasculature [103].

### Improvement of Cell Survival and Potency

In vivo after MI, cells are exposed to harsh conditions, such as ischaemia, oxidative stress and inflammation, which limit their survival. Environmental preconditioning regimes, such as hypoxia [76, 104], heat shock [105] and hydrogen peroxide [106] treatment, have been proposed to prepare the cells for the harsh conditions present in the infarcted area or to improve their therapeutic potential [107]. Direct application of radical scavengers to the heart resulted in improved adhesion of MSCs and consequently reduced fibrosis and infarct area [108].

The molecular mechanisms of preconditioning regimes comprise anti-apoptotic signalling, reduction of reactive oxygen species (ROS) generation and survival signalling via, amongst others, the Akt pathway [109]. Overexpression of Akt in transplanted MSCs improved their post-transplantation viability and therapeutic efficacy, leading to improved LV function by paracrine protection of the cardiomyocytes [110]. Similarly, overexpression of an Akt activator, periostin, in MSCs improved MSC and CM survival post-implantation, maintained cardiac function and limited infarct size [111].

Strategies to manipulate the inflammatory environment post-MI have been proposed, as the local inflammatory milieu affects both the survival of transplanted cells and the adverse remodeling of the myocardium. Kang et al. [112] demonstrated that priming peripheral blood mononuclear cells (PBMCs) with the supernatant of activated platelets resulted in M2 polarization of macrophages, induced angiogenesis and exerted beneficial effects post-MI.

Finally, miRNA molecules have also been suggested for ameliorating the stem cell survival and retention limitation. Hu et al. [113] treated CPCs with pro-survival miRNAs (miR-21, miR-24 and miR-221) and reported increased survival in vitro under serum starvation and increased retention and reduced adverse cardiac remodelling post-MI in vivo. In addition, upregulation of miRNA-21 in Sca-1 positive CSCs resulted in increased migration and proliferation [114].

### Combinations of Cells

Since different cell types may have different mechanisms of action, the use of a combination of cells has been proposed. For example, CPCs may be superior for the formation of new CMs, whereas MSCs are known to secrete a variety of paracrine factors and have immunomodulatory potential [115], and EPCs contribute to blood vessel development and maturation [116].

Williams et al. [117] applied a combination of human MSCs and c-kit positive CSCs by transepicardial injection in a swine MI model. The combination of both cell types resulted in a 2-fold-greater reduction in scar size compared with either cell type administered alone. This was paralleled by an enhanced recovery of both systolic and diastolic function.

Alternatively, 3D CardioClusters comprising CPCs, MSCs and EPCs and stem cell hybrids of MSCs and CPCs—CardioChimeras—have also been proposed [118].

## Alternative Therapeutic Strategies

Direct regeneration of the myocardium in the clinical setting remains elusive. Manipulation of paracrine interactions may be more feasible although this has been limited by poor control of delivery, wash-out or degradation. Therapeutic angiogenesis by application of growth factors such as VEGF may have failed to demonstrate clinical benefit due to the difficulty of maintaining the local VEGF concentration at an effective level [119]. Biomaterials could be devised for cardiac delivery of angiogenic and cardioprotective agents with controllable release kinetics. In addition, local stem cell pools could be activated or recruited by application of paracrine agents, such as IGF-l [120] and Fstl1 [121].

Cardiomyocytes or progenitors can be delivered within a scaffold such as in situ polymerizable hydrogels and pre-cast scaffolds, to immobilize cells in the area in which they are required [122] (Fig. 2). Indeed, Araña et al. [123] could recover 25.3±7.0 % of cells seeded in scaffolds, 1 week after cell transplantation, whereas in cell-injected control animals, no cells could be recovered. Material and chemical properties of the scaffolds play dominant roles in biocompatibility, engraftment/rejection and cardiac remodelling. This is beyond the scope of this review and has been covered elsewhere [124].

Finally, as a novel therapeutic strategy, the adult heart contains a significant proportion of cardiac fibroblasts, which can be targeted for direct reprogramming to cardiomyocytes or to cardiac progenitor cells without the need of the intermediate iPS step [125]. However, further optimization is required given the low efficacy and technical difficulty of this technique [126].

## Conclusions

SCT in the heart is at a crucial standpoint; do we continue to persist with cell therapy despite the barrage of difficulties that arise or is it time to concentrate our efforts towards alternative approaches? It can be said that the emergence of the paracrine hypothesis fuels the latter argument, if stem cells merely offer a means to enhance endogenous repair and regenerative mechanisms. Notwithstanding, SCT clearly has benefits beyond this narrow view including (a) stem cells have homing properties enabling them to target sites of injury more efficiently than protein based or genetic approaches; (b) the release of cytokines and growth factors from stem cells is a controlled process dependent on feedback and paracrine relationships with other cells, which ensures that specific factors in specific combinations target specific cells at specific times, a feat difficult to achieve with other therapies; and crucially, (c) the potential of more pluripotent stem cells to form new cardiomyocytes that can replace and regenerate large areas of the myocardium continues to offer a curative solution for end-stage HF. With this more holistic approach in mind, sustained interest and attention to overcome the current limitations of cell therapy will continue to be the priority of research in this field.

## Abbreviations

ALCADIA: Autologous human cardiac-derived stem cell to treat ischemic cardiomyopathy
AMI: Acute myocardial infarction
bFGF: Basic fibroblast growth factor
BM: Bone marrow
BM-MSC: Bone marrow-derived mesenchymal stem cell
CADUCEUS: CArdiosphere-Derived aUtologous stem CElls to reverse ventricUlar dySfunction
CDC: Cardiosphere-derived stem cell
CM: Cardiomyocyte
CPC: Cardiac progenitor cell
CSC: Cardiac stem cell
CVD: Cardiovascular disease
CXCR4: C-X-C chemokine receptor 4
EF: Ejection fraction
EPC: Endothelial progenitor cell
EPO: Erythropoietin
ES: Embryonic stem cell
ES-CM: Embryonic stem cell-derived cardiomyocyte
HF: Heart failure
HGF: Hepatocyte growth factor
HIF: Hypoxia-inducible factor
HLHS: Hypoplastic left heart syndrome
I/R: Ischaemia/reperfusion
IC: Intracoronary
IGF-1: Insulin-like growth factor-1
IL-10: Interleukin-10
IL-6: Interleukin-6
IM: Intramyocardial
iPS: Induced pluripotent stem cell
IV: Intravenous
LV: Left ventricle
MI: Myocardial infarction
miRNA: Micro RNA
MSC: Mesenchymal stem cell
PBMC: Peripheral blood mononuclear cell
PI3K: Phosphoinositide 3-kinase
ROS: Reactive oxygen species
RVEF: Right ventricular ejection fraction
SCIPIO: Stem Cell Infusion in Patients with Ischemic cardiOmyopathy
SCT: Stem cell therapy
SDF-1: Stromal cell-derived factor-1
SP: Side population
TICAP: Transcoronary infusion of cardiac progenitor cells in patients with single ventricle physiology
VEGF: Vascular endothelial growth factor
